# Supplementary material for: Role of Dietary Fibre in Managing Periodontal Diseases—A Systematic Review and Meta-Analysis of Human Intervention Studies
Source: Nutrients. 2023 Sep 18;15(18):4034. doi: 10.3390/nu15184034 (PMC10536522; doi:10.3390/nu15184034)
Supplement: Supplementary file 1 [file nutrients-15-04034-s001.zip › nutrients-2599039-supplementary.pdf]

Supplementary Table S1: Electronic search strategy.

Database	Keywords	Search level	Studies identified
MEDLINE	((((((((((((((((((((((Intervention*[MeSH Terms]) OR (Intervention* [Title/Abstract])) OR (RCT [Title/Abstract])) OR (RCT [MeSH Terms])) OR (Randomi?ed controlled trial* [MeSH Terms])) OR (Randomi?ed controlled trial* [Title/Abstract])) OR (Cross over [Title/Abstract])) OR (Cross over [MeSH Terms])) OR (Clinical trial* [MeSH Terms])) OR (Clinical trial* [Title/Abstract])) OR (Sequential feeding trial* [Title/Abstract])) OR (Sequential feeding trial* [MeSH Terms])) OR (Parallel [MeSH Terms])) OR (Parallel [Title/Abstract])) AND (((Oral disease* [MeSH Terms]) OR (Oral health [MeSH Terms])) OR (periodont* disease* [MeSH Terms])) OR (periodont* health [MeSH Terms])) OR (Dental disease* [MeSH Terms])) OR (Gum* disease* [MeSH Terms])) OR (Gingi* disease* [MeSH Terms])) OR (Tooth disease* [MeSH Terms])) OR (Tooth health [MeSH Terms])) OR (Teeth disease* [MeSH Terms])) OR (Teeth health [MeSH Terms])) OR (Mouth disease* [MeSH Terms])) OR (Dental Caries [MeSH Terms])) OR (tooth Caries [MeSH Terms])) OR (oral Caries [MeSH Terms])) OR (teeth Caries [MeSH Terms])) OR (Dental Demineralі?ation [MeSH Terms])) OR (tooth Demineralі?ation [MeSH Terms])) OR (oral Demineralі?ation [MeSH Terms])) OR (teeth Demineralі?ation [MeSH Terms])) OR (Dental Decay* [MeSH Terms])) OR (tooth Decay* [MeSH Terms])) OR (oral Decay*[MeSH Terms])) OR (teeth Decay* [MeSH Terms])) OR (Dental Cavit* [MeSH Terms])) OR (tooth Cavit* [MeSH Terms])) OR (oral Cavit* [MeSH Terms])) OR (teeth Cavit*[MeSH Terms])) OR (Dental Cario* [MeSH Terms])) OR (tooth Cario* [MeSH Terms])) OR (oral Cario* [MeSH Terms])) OR (teeth Cario*[MeSH Terms])) OR ("oral hygiene" index* [MeSH Terms])) OR ("oral hygiene" scale* [MeSH Terms])) OR ("orthodontic treatment need" index* [MeSH Terms])) OR ("orthodontic treatment need" scale* [MeSH Terms])) OR (Periodontal index*[MeSH Terms])) OR (Periodontal scale* [MeSH Terms])) OR (Plaque index* [MeSH Terms])) OR (Plaque scale* [MeSH Terms])) OR ("significant caries" index* [MeSH Terms])) OR ("significant caries" scale* [MeSH Terms])) AND ((((((((((((((Fibre*[MeSH Terms]) OR (prebiotic*[MeSH Terms])) OR (resistant starch[MeSH Terms])) OR (pectin[MeSHTerms])) OR (cellulose*[MeSH Terms])) OR (lignin[MeSH Terms])) OR (gum[MeSH Terms]))	Advanced search in article Title, Abstract, MeSH terms level	3656

	OR (mucilage[MeSH Terms])) OR (roughage[MeSH Terms])) OR (Hemicellulose*[MeSH Terms])) OR (psyllium[MeSH Terms])) OR (beta-glucan*[MeSH Terms])) OR (Fiber*[MeSH Terms])) OR (inulin[MeSH Terms])) OR ((((((((((((((Fibre* [Title/Abstract]) OR (prebiotic* [Title/Abstract])) OR (resistant starch [Title/Abstract])) OR (pectin[Title/Abstract])) OR (cellulose* [Title/Abstract])) OR (lignin [Title/Abstract])) OR (gum [Title/Abstract])) OR (mucilage [Title/Abstract])) OR (roughage [Title/Abstract])) OR (Hemicellulose* [Title/Abstract])) OR (psyllium[Title/Abstract])) OR (beta-glucan*[Title/Abstract])) OR (Fiber*[Title/Abstract])) OR (inulin [Title/Abstract])))) Filters: Humans, English		
SciVerse Scopus®	((TITLE-ABS-KEY (oral AND disease*) OR TITLE-ABS-KEY (oral AND health) OR TITLE-ABS-KEY (periodont* AND disease*) OR TITLE-ABS-KEY (periodont* AND health) OR TITLE-ABS-KEY (dental AND disease*) OR TITLE-ABS-KEY (gum* AND disease*) OR TITLE-ABS-KEY (gingi* AND disease*) OR TITLE-ABS-KEY (tooth AND disease*) OR TITLE-ABS-KEY (tooth AND health) OR TITLE-ABS-KEY (teeth AND disease*) OR TITLE-ABS-KEY (teeth AND health) OR TITLE-ABS-KEY (mouth AND disease*) OR TITLE-ABS-KEY (dental AND caries) OR TITLE-ABS-KEY (tooth AND caries) OR TITLE-ABS-KEY (oral AND caries) OR TITLE-ABS-KEY (teeth AND caries) OR TITLE-ABS-KEY (dental AND demineralі?ation) OR TITLE-ABS-KEY (tooth AND demineralі?ation) OR TITLE-ABS-KEY (oral AND demineralі?ation) OR TITLE-ABS-KEY (teeth AND demineralі?ation) OR TITLE-ABS-KEY (dental AND decay*) OR TITLE-ABS-KEY (tooth AND decay*) OR TITLE-ABS-KEY (oral AND decay*) OR TITLE-ABS-KEY (teeth AND decay*) OR TITLE-ABS-KEY (dental AND cavit*) OR TITLE-ABS-KEY (tooth AND cavit*) OR TITLE-ABS-KEY (oral AND cavit*) OR TITLE-ABS-KEY (teeth AND cavit*) OR TITLE-ABS-KEY (dental AND cario*) OR TITLE-ABS-KEY (tooth AND cario*) OR TITLE-ABS-KEY (oral AND cario*) OR TITLE-ABS-KEY (teeth AND cario*) OR TITLE-ABS-KEY ("oral hygiene"index*) OR TITLE-ABS-KEY ("oral hygiene"scale*) OR TITLE-ABS-KEY ("orthodontic treatment need"index*) OR TITLE-ABS-KEY ("orthodontic treatment need"scale*) OR TITLE-ABS-KEY (periodontal AND index*) OR TITLE-ABS-KEY (periodontal AND scale*) OR TITLE-ABS-KEY (plaque AND index*) OR TITLE-ABS-KEY (plaque AND scale*) OR TITLE-ABS-KEY ("significant caries"index*) OR TITLE-ABS-KEY ("significant caries"scale*))) AND ((TITLE-ABS-KEY (fibre*) OR	Article title, Abstract, Keywords	4608

	TITLE-ABS-KEY (prebiotic*) OR TITLE-ABS-KEY (resistant AND starch) OR TITLE-ABS-KEY (pectin) OR TITLE-ABS-KEY (cellulose*) OR TITLE-ABS-KEY (lignin) OR TITLE-ABS-KEY (gum) OR TITLE-ABS-KEY (mucilage) OR TITLE-ABS-KEY (roughage) OR TITLE-ABS-KEY (hemicellulose*) OR TITLE-ABS-KEY (psyllium) OR TITLE-ABS-KEY (beta-glucan*) OR TITLE-ABS-KEY (fiber*) OR TITLE-ABS-KEY (inulin))) AND ((TITLE-ABS-KEY (intervention*) OR TITLE-ABS-KEY (rct) OR TITLE-ABS-KEY (randomi?ed AND controlled AND trial*) OR TITLE-ABS-KEY (cross AND over) OR TITLE-ABS-KEY (clinical AND trial*) OR TITLE-ABS-KEY (sequential AND feeding AND trial*) OR TITLE-ABS-KEY (parallel))) AND (LIMIT-TO (LANGUAGE, "English")) AND (LIMIT-TO (EXACTKEYWORD, "Human") OR LIMIT-TO (EXACTKEYWORD, "Humans")) AND (LIMIT-TO (DOCTYPE, "ar"))		
Web of Science®	(((Oral disease* (Abstract) or Oral health (Abstract) or periodont* disease* (Abstract) or periodont* health (Abstract) or Dental disease* (Abstract) or Gum* disease* (Abstract) or Gingi* disease* (Abstract) or Tooth disease* (Abstract) or Tooth health (Abstract) or Teeth disease* (Abstract) or Teeth health (Abstract) or Mouth disease* (Abstract) or Dental Caries (Abstract) or tooth Caries (Abstract) or oral Caries (Abstract) or teeth Caries (Abstract) or Dental Demineral?ation (Abstract) or tooth Demineral?ation (Abstract) or oral Demineral?ation (Abstract) or teeth Demineral?ation (Abstract) or Dental Decay* (Abstract) or tooth Decay* (Abstract) or oralDecay* (Abstract) or teeth Decay* (Abstract) or Dental Cavit* (Abstract) or tooth Cavit* (Abstract) or oral Cavit* (Abstract) or teeth Cavit* (Abstract) or Dental Cario* (Abstract) or tooth Cario* (Abstract) or oral Cario* (Abstract) or teeth Cario* (Title) or "oral hygiene" index* (Title) or "oral hygiene" scale* (Title) or "orthodontic treatment need" index* (Title) or "orthodontic treatment need" scale* (Abstract) or Periodontal index* (Abstract) or Periodontal scale* (Abstract) or Plaque index* (Abstract) or Plaque scale* (Abstract) or "significant caries" index* (Abstract) or "significant caries" scale* (Abstract)) OR Oral disease* (Title) or Oral health (Title) or periodont* disease* (Title) or periodont* health (Title) or Dental disease* (Title) or Gum* disease* (Title) or Gingi* disease* (Title) or Tooth disease* (Title) or Tooth health (Title) or Teeth disease* (Title) or Teeth health (Title) or Mouth disease* (Title) or Dental Caries (Title) or tooth Caries (Title) or oral Caries (Title) or teeth Caries (Title) or Dental Demineral?ation (Title) or tooth Demineral?ation	Advanced search operator "Topic Search" (Title and Abstract)	1385

	(Title) or oral Demineraliʔation (Title) or teeth Demineraliʔation (Title) or Dental Decay* (Title) or tooth Decay* (Title) or oral Decay* (Title) or teeth Decay* (Title) or Dental Cavit* (Title) or tooth Cavit* (Title) or oral Cavit* (Title) or teeth Cavit* (Title) or Dental Cario* (Title) or tooth Cario* (Title) or oral Cario* (Title) or teeth Cario* (Title) or "oral hygiene" index* (Title) or "oral hygiene" scale* (Title) or "orthodontic treatment need" index* (Title) or "orthodontic treatment need" scale* (Title) or Periodontal index* (Title) or Periodontal scale* (Title) or Plaque index* (Title) or Plaque scale* (Title) or "significant caries" index* (Title) or "significant caries" scale* (Title)) AND Fibre* (Title) or prebiotic* (Title) or resistant starch (Title) or pectin (Title) or cellulose* (Title) or lignin (Title) or gum (Title) or mucilage (Title) or roughage (Title) or Hemicellulose* (Title) or psyllium (Title) or beta-glucan* (Title) or Fiber* (Title) or inulin (Title) or Fibre* (Abstract) or prebiotic* (Abstract) or resistant starch (Abstract) or pectin (Abstract) or cellulose* (Abstract) or lignin (Abstract) or gum (Abstract) or mucilage (Abstract) or roughage (Abstract) or Hemicellulose* (Abstract) or psyllium (Abstract) or beta-glucan* (Abstract) or Fiber* (Abstract) or inulin (Abstract)) AND Intervention* (Title) or RCT (Title) or Randomiʔed controlled trial* (Title) or Cross over (Title) or Clinical trial* (Title) or Sequential feeding trial* (Title) or Parallel (Title) or Intervention* (Abstract) or RCT (Abstract) or Randomiʔed controlled trial* (Abstract) or Cross over (Abstract) or Clinical trial* (Abstract) or Sequential feeding trial* (Abstract) or Parallel (Abstract) and English (Languages)		
CINAHL	S1 (MH "Periodontitis+") OR "periodontitis" S2 "periodont* health" S3 (MH "Periodontal Diseases+") OR "periodont* disease*" S4 "Dental disease*" S5 (MH "Gingival Diseases+") OR "Gum disease*" S6 "Gingi* disease*" S7 (MH "Tooth Diseases+") OR "Tooth disease*" S8 "Tooth health" S9 "Bone loss*" S10 (MH "Chronic Periodontitis") OR "Chronic Periodontitis" S11 "Periodontal inflammat*" S12 (MH "Gingivitis+") OR "Gingivitis" S13 "Gingival Inflammat*" S14 (MH "Aggressive Periodontitis") OR "Aggressive periodontitis" S15 "Pocket depth" S16 (MH "Periodontal Attachment Loss") OR "Periodontal Attachment Loss*" S17 "Clinical attachment loss*" S18 "Bleeding on probing" S19 "Clinical probing depth" S20 "Gingival bleed*" S21 (MH "Periodontal Ligament") OR "Periodontal ligament*" S22 "Gingival crevicular fluid*" S23 "porphyromonasgingivalis" S24 (MH "Periodontal	Advanced search operator "Topic Search" (Title and Abstract)	2313

	Atrophy+") OR "Periodontal Atrophy" S25 (MH "Gingival Recession") S26 (MH "Alveolar Bone Loss") S27 "periodont* pathogen*" S28 "dental plaque" S29 (MH "Gingiva+") OR "gingiv*" S68 "Gingival Recession" S69 "Alveolar bone loss" S70 (MH "Dental Plaque") S74 (MH "Periodontal Pocket") OR "periodontal pocket" S75 (MH "Periodontal Abscess") OR "periodontal abscess"<br><br>S30 "Fibre" S31 "Fiber" S32 "Fibrous" S33 (MH "Prebiotics") S34 "Inulin" S35 (MH "Resistant Starch") OR "resistant starch" S36 "pectin*" S37 (MH "Cellulose") OR "cellulose*" S38 "lignin" S39 "gum" S40 "Plant Mucilage" S41 "roughage" S42 "Hemicellulose*" S43 (MH "Psyllium") OR "psyllium" S44 (MH "Beta-Glucans") OR "beta-Glucans" S45 "Whole grain*" S46 "Bran" S47 "Crude fibre" S48 "soluble fibre" S49 "fermentable fibre" S50 "insoluble fibre" S51 "Dextrin*" S52 (MH "Oligosaccharides+") S53 "oligosaccharide*" S54 "wheat bran" S55 (MH "Polysaccharides+") OR "polysaccharide*" S56 "non-starch polysaccharide*" S57 "arabinoxylan" S58 "fructan*" S59 "polyuronide" S60 "polyuronide" S61 "raffinose" S62 "polydextrose" S63 "xanthan gum" S64 "guar gum" S65 (MH "Plant Gums+") OR "Plant gum*" S66 "Methylcellulose" S67 "Arabinogalactan" S72 "prebiotic*" S73 "galactomannan"<br><br>S71 "human*" OR (MH "Human+"): 258,728<br><br>Perio: 68,482<br>S76 S1 OR S2 OR S3 OR S4 OR S5 OR S6 OR S7 OR S8 OR S9 OR S10 OR S11 OR S12 OR S13 OR S14 OR S15 OR S16 OR S17 OR S18 OR S19 OR S20 OR S21 OR S22 OR S23 OR S24 OR S25 OR S26 OR S27 OR S28 OR S29 OR S68 OR S69 OR S70 OR S74 OR S75<br><br>Fibre: 69,207<br>S77 S30 OR S31 OR S32 OR S33 OR S34 OR S35 OR S36 OR S37 OR S38 OR S39 OR S40 OR S41 OR S42 OR S43 OR S44 OR S45 OR S46 OR S47 OR S48 OR S49 OR S50 OR S51 OR S52 OR S53 OR S54 OR S55 OR S56 OR S57 OR S58 OR S59 OR S60 OR S61 OR S62 OR S63 OR S64 OR S65 OR S66 OR S67 OR S72 OR S73<br>S78 S71 AND S76 AND S77		
EMBASE	1. Periodontitis.mp. or periodontitis/ 2. periodont* health.mp. 3. periodontal disease/ or periodont* disease.mp. 4. Dental disease*.mp. 5. Gum disease*.mp. 6. gingiva disease/ or Gingi* disease*.mp. 7. Tooth disease.mp. or tooth disease/ 8. Tooth health.mp. 9. Bone loss*.mp.10 Chronic Periodontitis.mp. or chronic	Advanced search operator "Topic Search"	5960

	periodontitis/ 11. Periodontal inflammat*.mp.12. Gingivitis.mp. or gingivitis/ 13. Gingival Inflammat*.mp. 14. Aggressive periodontitis.mp. or aggressive periodontitis/ 15. Pocket depth.mp. 16. Periodontal Attachment Loss*.mp. 17. Clinical attachment loss*.mp. 18. "Bleeding on probing".mp. 19. Clinical probing depth.mp. 20. gingiva bleeding/ or Gingival bleed*.mp. 21. periodontal ligament/ or Periodontal ligament*.mp. 22. Gingival crevicular fluid*.mp. 23. Porphyromonas gingivalis.mp. or Porphyromonasgingivalis/ 24. Periodontal Atrophy.mp. 25. Gingival Recession.mp. 26. Alveolar Bone Loss.mp. or alveolar bone loss/ 62. periodont* pathogen*.mp. 63. dental plaque.mp. or tooth plaque/ 64. gingiva disease/ or gingiva/ or gingivitis/ or gingiv*.mp. 71. periodontal pocket.mp. or periodontal pocket/ 72. periodontal abscess.mp. or periodontal abscess/<br><br>27. Fibre.mp. 28. Fiber.mp. or dietary fiber/ or fiber/29. Fibrous.mp. 30. prebiotic*.mp. 31. inulin/ or Inulin.mp. 32. resistant starch.mp. 33. pectin/ or pectin*.mp. 34. cellulose*.mp. or cellulose/ 35. lignin/ or lignin.mp. 36. Gum.mp. 37. Plant Mucilage.mp. or mucilage/ 38. roughage.mp. or roughage/ 39. hemicellulose/ or Hemicellulose*.mp. 40 psyllium.mp. or ispagula/ 41. beta-Glucans.mp. or beta glucan/ 42. Whole grain*.mp. or whole grain/ 43. bran/ or Bran.mp. 44 hemicellulose/ or Hemicellulose.mp. 45. Crude fibre.mp. 46. soluble fibre.mp. 47. fermentable fibre.mp. 48. insoluble fibre.mp. 49. dextrin/ or Dextrin*.mp. 50. oligosaccharide/ or oligosaccharide*.mp. 51 wheat bran.mp. or wheat bran/ 52. polysaccharide/ or polysaccharide*.mp. 53. non-starch polysaccharide*.mp. 54. arabinoxylan.mp. or arabinoxylan/ 55. fructan/ or fructan*.mp. 56. Polyuronide.mp. 57. raffinose.mp. or raffinose/ 58. Polydextrose.mp. 59. xanthan gum.mp. 60. guar gum.mp. or guar gum/ 61. plant gum/ or Plant gum*.mp. 73. methylcellulose.mp. or methylcellulose/ 74. arabinogalactan.mp. or arabinogalactan/ 75. galactomannan.mp. or galactomannan/<br><br>Human: 6198265<br>69. human/ or human*.mp.<br><br>Perio: 223,788<br>76. 1 or 2 or 3 or 4 or 5 or 6 or 7 or 8 or 9 or 10 or 11 or 12 or 13 or 14 or 15 or 16 or 17 or 18 or 19 or 20 or 21 or 22 or 23 or 24 or 25 or 26 or 62 or 63 or 64 or 71 or 72	(Title and Abstract)	

	Fibre: 872,460<br>77. 27 or 28 or 29 or 30 or 31 or 32 or 33 or 34 or 35 or 36 or 37 or 38 or 39 or 40 or 41 or 42 or 43 or 44 or 45 or 46 or 47 or 48 or 49 or 50 or 51 or 52 or 53 or 54 or 55 or 56 or 57 or 58 or 59 or 60 or 61 or 73 or 74 or 75<br><br>Combined with AND: 1,868<br>78. 69 and 76 and 77		
Clinical trials gov.	(((Oral disease* (Abstract) or Oral health (Abstract) or periodont* disease* (Abstract) or periodont* health (Abstract) or Dental disease* (Abstract) or Gum* disease* (Abstract) or Gingi* disease* (Abstract) or Tooth disease* (Abstract) or Tooth health (Abstract) or Teeth disease* (Abstract) or Teeth health (Abstract) or Mouth disease* (Abstract) or Dental Caries (Abstract) or tooth Caries (Abstract) or oral Caries (Abstract) or teeth Caries (Abstract) or Dental Demineralı?ation (Abstract) or tooth Demineralı?ation (Abstract) or oral Demineralı?ation (Abstract) or teeth Demineralı?ation (Abstract) or Dental Decay* (Abstract) or tooth Decay* (Abstract) or oralDecay* (Abstract) or teeth Decay* (Abstract) or Dental Cavit* (Abstract) or tooth Cavit* (Abstract) or oral Cavit* (Abstract) or teeth Cavit* (Abstract) or Dental Cario* (Abstract) or tooth Cario* (Abstract) or oral Cario* (Abstract) or teeth Cario* (Title) or "oral hygiene" index* (Title) or "oral hygiene" scale* (Title) or "orthodontic treatment need" index* (Title) or "orthodontic treatment need" scale* (Abstract) or Periodontal index* (Abstract) or Periodontal scale* (Abstract) or Plaque index* (Abstract) or Plaque scale* (Abstract) or "significant caries" index* (Abstract) or "significant caries" scale* (Abstract)) OR Oral disease* (Title) or Oral health (Title) or periodont* disease* (Title) or periodont* health (Title) or Dental disease* (Title) or Gum* disease* (Title) or Gingi* disease* (Title) or Tooth disease* (Title) or Tooth health (Title) or Teeth disease* (Title) or Teeth health (Title) or Mouth disease* (Title) or Dental Caries (Title) or tooth Caries (Title) or oral Caries (Title) or teeth Caries (Title) or Dental Demineralı?ation (Title) or tooth Demineralı?ation (Title) or oral Demineralı?ation (Title) or teeth Demineralı?ation (Title) or Dental Decay* (Title) or tooth Decay* (Title) or oral Decay* (Title) or teeth Decay* (Title) or Dental Cavit* (Title) or tooth Cavit* (Title) or oral Cavit* (Title) or teeth Cavit* (Title) or Dental Cario* (Title) or tooth Cario* (Title) or oral Cario* (Title) or teeth Cario* (Title) or "oral hygiene" index* (Title) or "oral hygiene" scale* (Title) or "orthodontic treatment need" index* (Title) or	Advanced search operator "Topic Search" (Title and Abstract)	954

	"orthodontic treatment need" scale* (Title) or Periodontal index* (Title) or Periodontal scale* (Title) or Plaque index* (Title) or Plaque scale* (Title) or "significant caries" index* (Title) or "significant caries" scale* (Title)) AND Fibre* (Title) or prebiotic* (Title) or resistant starch (Title) or pectin (Title) or cellulose* (Title) or lignin (Title) or gum (Title) or mucilage (Title) or roughage (Title) or Hemicellulose* (Title) or psyllium (Title) or beta-glucan* (Title) or Fiber* (Title) or inulin (Title) or Fibre* (Abstract) or prebiotic* (Abstract) or resistant starch (Abstract) or pectin (Abstract) or cellulose* (Abstract) or lignin (Abstract) or gum (Abstract) or mucilage (Abstract) or roughage (Abstract) or Hemicellulose* (Abstract) or psyllium (Abstract) or beta-glucan* (Abstract) or Fiber* (Abstract) or inulin (Abstract)) AND Intervention* (Title) or RCT (Title) or Randomi?ed controlled trial* (Title) or Cross over (Title) or Clinical trial* (Title) or Sequential feeding trial* (Title) or Parallel (Title) or Intervention* (Abstract) or RCT (Abstract) or Randomi?ed controlled trial* (Abstract) or Cross over (Abstract) or Clinical trial* (Abstract) or Sequential feeding trial* (Abstract) or Parallel (Abstract) and English (Languages)		
Cochrane library	(((Oral disease* (Abstract) or Oral health (Abstract) or periodont* disease* (Abstract) or periodont* health (Abstract) or Dental disease* (Abstract) or Gum* disease* (Abstract) or Gingi* disease* (Abstract) or Tooth disease* (Abstract) or Tooth health (Abstract) or Teeth disease* (Abstract) or Teeth health (Abstract) or Mouth disease* (Abstract) or Dental Caries (Abstract) or tooth Caries (Abstract) or oral Caries (Abstract) or teeth Caries (Abstract) or Dental Demineraliz?ation (Abstract) or tooth Demineraliz?ation (Abstract) or oral Demineraliz?ation (Abstract) or teeth Demineraliz?ation (Abstract) or Dental Decay* (Abstract) or tooth Decay* (Abstract) or oralDecay* (Abstract) or teeth Decay* (Abstract) or Dental Cavit* (Abstract) or tooth Cavit* (Abstract) or oral Cavit* (Abstract) or teeth Cavit* (Abstract) or Dental Cario* (Abstract) or tooth Cario* (Abstract) or oral Cario* (Abstract) or teeth Cario* (Title) or "oral hygiene" index* (Title) or "oral hygiene" scale* (Title) or "orthodontic treatment need" index* (Title) or "orthodontic treatment need" scale* (Abstract) or Periodontal index* (Abstract) or Periodontal scale* (Abstract) or Plaque index* (Abstract) or Plaque scale* (Abstract) or "significant caries" index* (Abstract) or "significant caries" scale* (Abstract)) OR Oral disease* (Title) or Oral health (Title) or periodont* disease* (Title) or periodont* health (Title) or Dental disease*	Advanced search operator "Topic Search" (Title and Abstract)	1095

	(Title) or Gum* disease* (Title) or Gingi* disease* (Title) or Tooth disease* (Title) or Tooth health (Title) or Teeth disease* (Title) or Teeth health (Title) or Mouth disease* (Title) or Dental Caries (Title) or tooth Caries (Title) or oral Caries (Title) or teeth Caries (Title) or Dental Demineraliz?ation (Title) or tooth Demineraliz?ation (Title) or oral Demineraliz?ation (Title) or teeth Demineraliz?ation (Title) or Dental Decay* (Title) or tooth Decay* (Title) or oral Decay* (Title) or teeth Decay* (Title) or Dental Cavit* (Title) or tooth Cavit* (Title) or oral Cavit* (Title) or teeth Cavit* (Title) or Dental Cario* (Title) or tooth Cario* (Title) or oral Cario* (Title) or teeth Cario* (Title) or "oral hygiene" index* (Title) or "oral hygiene" scale* (Title) or "orthodontic treatment need" index* (Title) or "orthodontic treatment need" scale* (Title) or Periodontal index* (Title) or Periodontal scale* (Title) or Plaque index* (Title) or Plaque scale* (Title) or "significant caries" index* (Title) or "significant caries" scale* (Title)) AND Fibre* (Title) or prebiotic* (Title) or resistant starch (Title) or pectin (Title) or cellulose* (Title) or lignin (Title) or gum (Title) or mucilage (Title) or roughage (Title) or Hemicellulose* (Title) or psyllium (Title) or beta-glucan* (Title) or Fiber* (Title) or inulin (Title) or Fibre* (Abstract) or prebiotic* (Abstract) or resistant starch (Abstract) or pectin (Abstract) or cellulose* (Abstract) or lignin (Abstract) or gum (Abstract) or mucilage (Abstract) or roughage (Abstract) or Hemicellulose* (Abstract) or psyllium (Abstract) or beta-glucan* (Abstract) or Fiber* (Abstract) or inulin (Abstract)) AND Intervention* (Title) or RCT (Title) or Randomi?ed controlled trial* (Title) or Cross over (Title) or Clinical trial* (Title) or Sequential feeding trial* (Title) or Parallel (Title) or Intervention* (Abstract) or RCT (Abstract) or Randomi?ed controlled trial* (Abstract) or Cross over (Abstract) or Clinical trial* (Abstract) or Sequential feeding trial* (Abstract) or Parallel (Abstract) and English (Languages)		

Supplementary Table S2: List of excluded studies.

**Wrong Population (9)**
1. Effect of sugar-free chewing gum on plaque and gingivitis among 14-15-year-old school children: A randomized controlled trial
2. Effect of xylitol gum on the level of oral mutans streptococci of preschoolers: Block-randomised trial
3. Actinobacillus actinomycetemcomitans Y4 capsular polysaccharide induces IL-1β mRNA expression through the JNK pathway in differentiated THP-1 cells
4. The effect of raisin-containing cereals on the pH of dental plaque in young children
5. Effect of xylitol on dental caries and salivary Streptococcus mutans levels among a group of mother-child pairs
6. Effect of the Use of Xylitol Gum in the Prevention of Caries Lesions in Children Living in Ladakh
7. Thirty-nine-month xylitol chewing-gum programme in initially 8-year-old school children: a feasibility study focusing on mutans streptococci and lactobacilli
8. Properties of whole saliva and dental plaque in relation to 40-month consumption of chewing gums containing xylitol, sorbitol of sucrose
9. Effects of maltitol and xylitol chewing-gums on parameters involved in dental caries development
**Wrong Intervention (51)**
1. Effectiveness of rinsing water and chewing gum on restoring pH of saliva after drinking the drinking yogurt
2. Effect of a sugar-free chewing gum containing magnolia bark extract on different variables related to caries and gingivitis: A randomized controlled intervention trial
3. Xylitol gum and maternal transmission of mutans streptococci
4. Oxidized cellulose mesh II. Using hydroxy-apatite bone grafting material in the treatment of infrabony defects
5. Effect of maltitol-containing chewing gum use on the composition of dental plaque microbiota in subjects with active dental carie
6. Probiotics for gum health during treatment with braces
7. Remineralisation by chewing sugar-free gums in a randomised, controlled in situ trial including dietary intake and gauze to promote plaque formation

8.	The Effect of Probiotic Supplementation in the form of Mouthwash with Scaling and Root planing on the Periodontal Indeces in Patients with Generalized Chronic Periodontitis
9.	Comparison of the effect of caseinphosphopeptide-amorphous calcium phosphate (CPP-ACP) and Xylitol Chewing gums and probiotic yogurt on streptocuccos mutans and microbial dental plaque
10.	Effect of xylitol-containing chewing gum on sorbitol metabolism in dental plaque
11.	Effect of sucrose concentration on dental biofilm formed in situ and on enamel demineralization
12.	Recognition of the carbohydrate modifications to the RgpA protease of Porphyromonas gingivalis by periodontal patient serum IgG
13.	Cementogenic potential of multipotential mesenchymal stem cells purified from the human periodontal ligament
14.	Preparation and evaluation of β-glucan hydrogel prepared by the radiation technique for drug carrier applications
15.	Pectin-like acidic polysaccharide from Panax ginseng with selective antiadhesive activity against pathogenic bacteria
16.	Effects of probiotic consumption on gingival inflammation
17.	The effect of two types of chewing gum containing CPP - ACP and xylitol on caries-causing bacteria in the mouth
18.	Ethylcellulose inserts of an orphan drug for periodontitis: Preparation, in vitro, and clinical studies
19.	Regulation of fructosyltransferase activity by carbohydrates, in solution and immobilized on hydroxyapatite surfaces
20.	The effect of chlorhexidine acetate/xylitol chewing gum on the plaque and gingival indices of elderly occupants in residential homes
21.	Relationship between carbohydrate intake and polysaccharide-storing micro-organisms in dental plaque.
22.	Effects of short-term xylitol chewing gum on pro-inflammatory cytokines and Streptococcus mutans: a randomized, placebo-controlled trial
23.	The antibacterial effect of magnolia mouthwash on the levels of salivary streptococcus mutans in dental plaque: A randomized, single-blind, placebo-controlled trial
24.	High-intensity ultrasound-assisted formation of cellulose nanofiber scaffold with low and high lignin content and their cytocompatibility with gingival fibroblast cells

25. Can oral probiotics contribute to a well-balanced oral flora in orthodontic patients with brackets? A Comparison of Lactobacillus Reuteri Prodentis and Streptococcus Salivarius M 18
26. Extracellular polysaccharides and cariogenic activity in gnotobiotic conditions of lysogenic and cured Streptococci
27. The influence of pressure changes on the retentive force and coronal microleakage of different types of posts in endodontically treated teeth during simulated dives
28. Remineralizaron of enamel subsurface lesions by xylitol chewing gum containing funoran and calcium hydrogenphosphate
29. The Efficacy of a Chewing Gum Containing Phyllanthus emblica Fruit Extract in Improving Oral Health
30. Effects of sugar-free chewing gum sweetened with xylitol or maltitol on the development of gingivitis and plaque: A randomized clinical tria
31. Glycosaminoglycans in peri-implant sulcus fluid from implants placed in sinus-inlay bone grafts
32. Effects of oligofructose-enriched inulin on intestinal absorption of calcium and magnesium and bone turnover markers in postmenopausal women
33. Effect of xylitol and sorbitol in chewing-gums on mutans streptococci, plaque pH and mineral loss of ename
34. The efficacy of an anti-gingivitis chewing gum
35. The effect of tetracycline fiber therapy on β-glucuronidase and interleukin-1β in crevicular fluid
36. Effect of acemannan, a polysaccharide extracted from aloe vera gel, on periodontium regeneration in periodontitis patient, a randomized clinical study
37. Comparison of topical tacrolimus 0.1 % in pectin ointment with clobetasol 0.5% ointment in adults with moderate to severe desquamative gingivitis: a 4-week, randomized, double-blind clinical trial.
38. The study effects of quercusinfectoria on the oral environment in gingivitis patients
39. D-Tagatose effectively reduces the number of Streptococcus mutans and oral bacteria in healthy adult subjects: A chewing gum pilot study and randomized clinical trial
40. effect of probiotics on gum disease
41. Effects of Dietary Sucrose Levels on Extracellular Polysaccharide Metabolism of Human Dental Plaque

42. Effect of a propolis tablet on the saliva and the amount of adolescent dental plaque microbes comparing with the use of another tablet without propolis
43. Composition of plaque and saliva following use of an alpha-tricalcium-phosphate-containing chewing gum and a subsequent sucrose challenge
44. Efficacy of a probiotic and chlorhexidine mouth rinses: A short-term clinical study
45. Evaluation of fermented milk containing probiotic on dental enamel and biofilm: In situ study
46. Are Lactobacillus salivarius G60 and inulin more efficacious to treat patients with oral halitosis and tongue coating than the probiotic alone and placebo? A randomized clinical trial
47. Effects of Prebiotic Oral Healthcare Products as an Adjunct to Non-surgical Periodontal Therapy
48. Clinical outcomes using prebiotic and probiotic lozenges in non-surgical management of chronic periodontitis patients
49. Dietary supplementation with rice bran fermented with Lentinus edodes increases interferon- activity without causing adverse effects: a randomized, double-blind, placebo-controlled, parallel-group study
50. Inulin Supplementation During Sanative Therapy to Further Improve Periodontal Healing
51. Possible benefits from nutritional supplements during periodontitis (gum disease) treatment
**Wrong study design (31)**
1. The effects of chewing gum on the formation of plaque on the smooth surface theeth
2. Effectiveness of CPP -ACP Chewing gums
3. Considering the effect of a new chewing gum in preventing dental plaque formation
4. Xylitol carryover effects on salivary mutans streptococci after 13 months of chewing xylitol gum
5. The paradox: Bleeding presentation in antiphospholipid syndrome
6. Whole-grain and fiber intakes and periodontitis risk in men
7. Teucrium polium chewing gum effect on dental caries prevention
8. Probiotics use for gum disease treatment
9. Comparison of the effect of CCP-ACP & Xylitol chewing gum & probiotic yogurt on periodontal indices

10. Preliminary study, by chewing gum, to evaluate the changes on saliva component, oral hygiene and masticatory function
11. Evidence that the serotype b antigenic determinant of Actinobacillus actinomycetemcomitans Y4 resides in the polysaccharide moiety of lipopolysaccharide
12. Polysaccharides and the absence of glycogen in the gingiva from normal and diabetic patients
13. The immunodominant outer membrane antigen of Actinobacillus actinomycetemcomitans is located in the serotype-specific high-molecular-mass carbohydrate moiety of lipopolysaccharide
14. The potential of dental-protective chewing gum in oral health interventions
15. Oral prebiotics and the influence of environmental conditions in vitro
16. The potential benefits of sugar-free chewing gum on the oral health and quality of life of older people living in the community: A randomized controlled trial
17. Xylitol and Sorbitol Effects on the Oral Microbiome
18. Comparative evaluation of the effects of xylitol and sugar-free chewing gums on salivary and dental plaque pH in children
19. Xylitol-containing chewing gum for caries prevention in students with disabilities: A randomised trial
20. Effects of Pycnogenol chewing gum on oral malodor, gingivitis and plaque formation
21. Effect of repeated intake of a sugar free fluoride-containing chewing gum on acidogenicity and microbial composition of dental plaque
22. Assess the use of Gum-paint and Tablets applied over gums along with Cleaning in Diabetic patients with Gum Disease
23. To evaluate whether chewing gums containing useful bacteria reduce the number of cavity causing bacteria in mouth more than existing commercially available chewing gums or not
24. Dietary fiber intake and dental health status in urban, urban-marginal, and rural communities in central Mexico
25. Effects of Short-Chain Fatty Acids on Human Oral Epithelial Cells and the Potential Impact on Periodontal Diseases: A Systematic Review of In Vitro Studies
26. Short chain fatty acids, menaquinones and ubiquinones and their effects on the host
27. Primary versus secondary ITP in adults; A comparative analysis of clinical and labortoryattriburtes in newly diagnosed patients in an Asian population

28. Your diet matters to your teeth and gums.
29. The effect of maltitol sweetened chewing gum on the oral microbiology -RCT-
30. Turku sugar studies x: Occurrence of polysaccharide-forming streptococci and ability of the mixed plaque microbiota to ferment various carbohydrates
31. Association between functional dental state and dietary intake of Chinese vegetarian old age home residents
**Wrong outcomes (4)**
1. Soluble corn fiber increases bone calcium retention in postmenopausal women in a dose-dependent manner: A randomized crossover trial
2. Comparative efficacy of 1% alendronate gel and tetracycline fibers as an adjunct to scaling and root planing: a randomized control clinical trial
3. Effect of psyllium gum and wheat bran on spontaneous energy intake
4. Wheat bran fiber supplementation and bone loss among older people

Supplementary Table S3: Other outcomes of included studies.

Other outcomes		Bartha et al. (2022) [30]			Kondo et al. (2014) [31]			Rajaram et al. (2021) [33]			Woelber et al. (2016) [28]			Woelber et al. (2019) [29]		
		Intervention (I)	Control (C)	Inter-P-values (I vs C)	Intervention (I)	Control (C)	Inter-P-values (I vs C)	Intervention (I)	Control (C)	Inter-P-values (Ivs C)	Intervention (I)	Control (C)	Inter-P-values (I vs C)	Intervention (I)	Control (C)	Inter-P-values (I vs C)
BW	Baseline (BL)	79.01 ± 15.62	69.00 ±12.37	0.107	-	-	-	-	-	-	-	-	-	71.72 ±11.47	72.45 ±16.29	0.8882
	Follow-up (FU)	77.29 ± 17.00	69.37 ±12.34	0.112	-		-	-	-	-	-	-	-	70.19 ±10.96	72.95 ±15.86	0.5837
	Intra-P-value (BL vs FU)	0.025	0.312		-	-		-	-		-	-		0.007	0.310	
BMI	Baseline (BL)	24.8 ± 3.24	23.54 ± 2.83	0.349	26.7 ± 3.0	-	-	-	-	-	-	-	-	23.52 ± 2.31	23.0 ±2.06	0.5206
	Follow-up (FU)	24.21 ± 3.43	23.69 ± 3.00	0.6261	25.2 ± 2.7	-	-	-	-	-	-	-	-	23.32 ± 2.91	24.70 ± 4.94	0.3592
	Intra-P-value (BL vs FU)	0.011	0.290		<0.0001									0.007	0.249	
WC	Baseline (BL)	84.41 ± 10.1	79.94 ± 8.56	0.446	92.2 ± 8.8	-	-	-	-	-	-	-	-	-	-	
	Follow-up (FU)	83.17 ± 10.47	78.92 ± 8.00	0.1727	88.1 ± 7.0	-	-	-	-	-	-	-	-	-	-	
	Intra-P-value	0.001	0.160		<0.0001									-	-	

	(BL vs FU)															
hs-CRP	Baseline (BL)	-	-	-	0.55 (0.31-1.72)	-	-	-	-	-	-	-		0.73 ± 0.87	1.34 ± 2.12	0.3114
	Follow-up (FU)	-	-	-	0.39 (0.19-1.36)	-	-	-	-	-	-	-		1.00 ± 2.03	0.65 ± 0.88	0.5450
	Intra-P-value (BL vs FU)	-	-		0.038	-		-	-		-	-		0.647	0.132	
IL-6	Baseline (BL)	-	-	-	-	-	-	-	-	-	-	-	-	1.46 ± 0.81	1.65 ± 1.71	0.7003
	Follow-up (FU)	-	-	-	-	-	-	-	-	-	-	-	-	1.41 ± 0.78	1.15 ± 0.31	0.2403
	Intra-P-value (BL vs FU)	-	-		-	-		-	-					0.856	0.293	
TNF-α	Baseline (BL)	-	-	-	-	-	-	-	-	-	-	-	-	4.56 ± 1.44	4.51 ± 1.58	0.9285
	Follow-up (FU)	-	-	-	-	-	-	-	-	-	-	-	-	4.48 ± 1.41	4.74 ± 1.80	0.6630
	Intra-P-value (BL vs FU)	-	-		-	-		-	-		-	-		0.805	0.745	

BW: Body Weight; BMI: Body Mass Index; WC: Waist Circumference; hs-CRP: high sensitive C reactive protein; IL-6: interleukin-1beta; TNF-α: tumor necrosis factor-alpha.
